# Nose to brain delivery of melatonin lipidic nanocapsules as a promising post-ischemic neuroprotective therapeutic modality

**DOI:** 10.1080/10717544.2022.2104405

**Published:** 2022-07-27

**Authors:** Eman A. Bseiso, Sarah A. AbdEl-Aal, Maha Nasr, Omaima A. Sammour, Nabaweya A. Abd El Gawad

**Affiliations:** a Department of Pharmaceutics and Industrial Pharmacy, Faculty of Pharmacy, October 6 University, Giza Governorate, Egypt; b Pharmacology and Toxicology Division, Department of Pharmacy, KUT University College, Al Kut, Wasit52001, Iraq; c Department of Pharmaceutics and Industrial Pharmacy, Faculty of Pharmacy, Ain-Shams University, Cairo, Egypt; d Department of Pharmaceutics and Industrial Pharmacy, Faculty of Pharmacy, Cairo University, Egypt

**Keywords:** Lipid nanocapsules, melatonin, nose to brain delivery, stroke, brain ischemia

## Abstract

Ischemic stroke accounts for about 87% of all strokes, causing long-term disability in adults, and is the second leading cause of death worldwide. In search of new therapeutic modalities, the use of neuroprotective agents loaded in nanocarriers to be delivered by noninvasive means (i.e. via intranasal route) became a popular approach. In the current study, melatonin (MEL) was loaded in lipidic nanocapsules (LNCs) prepared using the phase inversion method, and characterized in terms of size, polydispersity, zeta potential, *in vitro* drug release, viscosity, storage stability, and *ex vivo* permeation across sheep nasal mucosa. Moreover, MEL-LNCs were tested for efficacy in cerebral ischemia/reperfusion (I/R/) injury model through histopathological assessment, and analysis of oxidative stress markers, pro-inflammatory cytokines, and apoptotic markers. Results showed that LNCs exhibited particle size ranging from 18.26 to 109.8 nm, negative zeta potential, good storage stability, spherical morphology, and a burst release followed by a sustained release pattern. LNCs exhibited 10.35 folds higher permeation of MEL than the drug solution across sheep nasal mucosa. Post-ischemic intranasal administration of MEL-LNCs revealed lowering of oxidative stress manifested by a decrease in malondialdehyde levels, and elevation of glutathione and superoxide dismutase levels, lowering of the inflammatory markers tumor necrosis factor-α, NO, myeloperoxidase, and significant inhibition of Caspase-3 activity as an apoptotic marker. Western blot analysis delineated a recovery of protein expression Nrf-2 and HO-1 with downregulation in the parent inflammatory markers nuclear factor kappa B p65, inducible nitric oxide synthase, Bax, and Cytochrome C expressions, and upregulation of B-cell lymphoma-2 Bcl-2, hence promoting neuronal survival. This was supported by histological evidence, revealing significant restoration of hippocampal neurons. In light of the above, it can be concluded that MEL-LNCs could be a promising delivery system for nose to brain delivery for treatment of cerebral ischemia.

## Introduction

Stroke is a serious mortality-causing disease **(**Gebreyohannes et al., 2019**)**. In 2017, there were 2.7 million deaths due to ischemic stroke **(**Benjamin et al., 2018**)**. A stroke occurs when the blood flow to an area of the brain is affected, causing brain cells to die and brain damage to occur **(**Gund et al., 2013**)**, with catastrophic consequences such as disabilities in speech, movement, or memory **(**Radak et al., 2017**)**.

According to previously published reports, the cerebral ischemia/reperfusion injury causes irreversible brain damage and neuronal death by complex processes, namely oxidative stress and inflammation **(**Wu et al., 2020**)**. Oxidative stress plays a main role in the development of the ischemic injury cascade, since the reactive oxygen species (ROS) that are produced during ischemia cause lipid peroxidation and inflammation, in which the latter alters the balance between pro-inflammatory and anti-inflammatory factors, leading to exacerbation of injury, and consequently neuronal and brain death **(**Wu et al., 2020**)**. Therefore, the use of a molecule with both antioxidant and anti-inflammatory properties can be a promising treatment modality for ischemic stroke.

Melatonin (MEL) is a neurohormone that was reported to exert notable antioxidant and anti-inflammatory properties, achieving its defense against several cerebral ischemia/reperfusion injury models **(**Reiter et al., 1996; Hardeland et al., 2006**)**. It also exhibits neuroprotective actions **(**Cheung, 2003; Altun & Ugur-Altun, 2007**)**. The neuroprotective mechanisms of MEL after cerebral ischemia include the inhibition of endothelin converting enzyme-1 **(**Kilic et al., 2004**)** and matrix metalloproteinase-9 **(**Kim & Lee 2014**)**, and enhanced MEK/ERK/p90RSK signaling cascade **(**Koh, 2008**)**. Despite its therapeutic merits, the use of MEL is hampered by its short half-life, and poor aqueous solubility (Hatem et al., 2018a,b**)**. Therefore, nanocarriers were introduced for MEL to overcome the aforementioned pharmaceutical limitations. MEL was formulated as nanostructured lipid carriers and aspasomes for topical treatment of androgenic alopecia **(**Hatem et al., 2018a,b**)**. In addition, MEL nanostructured lipid carriers were proven promising in treatment of infertility **(**Siahdasht et al., 2020**)**. Elastic liposomal system for transdermal delivery of MEL showed high permeation than the drug solution for potential antioxidant uses **(**Dubey et al., 2006**)**. MEL-loaded chitosan nanoparticles were used for the treatment of various types of cancer **(**Shokrzadeh & Ghassemi-Barghi, 2018**)**. Furthermore, MEL solid lipid nanoparticles showed protective effect from testicular trauma in rats **(**Mirhoseini et al., 2019**)**. MEL has been also used as a powerful endogenous antioxidant in treating ischemic heart tissue, and as an anti-glaucoma drug when loaded in poly-lactic-glycolic acid (PLGA) nanoparticles **(**Musumeci et al., 2013**;** Ma et al., 2018**)**. MEL PLGA nanoparticles also exhibited adjunctive effect when used in the treatment of osteosarcoma **(**Altındal & Gümüşderelioğlu, 2016**)**. MEL was also loaded in polycaprolactone nanoparticles for treatment of glioblastoma **(**de Oliveira Junior et al., 2019**)**. In addition, Wang et al. reported that MEL-selenium nanoparticles exhibited hepatoprotective effect on immunological liver injury in mice **(**Wang et al., 2005**)**.

Recently, intranasal administration has been proven promising in delivering therapeutic agents to the brain **(**Barakat et al., 2017; Nasr & Wahdan, 2019**)**. Nose-to-brain delivery has been investigated as a noninvasive means of providing direct access to the brain through the olfactory and trigeminal nerves. To improve the brain delivery of MEL via the intranasal route in the current study, it was loaded in lipid nanocapsules (LNCs), to increase its nasal permeability and control its release. LNCs are surfactant-shelled nanosystems **(**Nasr & Abdel-Hamid, 2015**)**, which have been proven promising in delivering therapeutic moieties to the brain using the intranasal route **(**Mohsen et al., 2020**)**.

Therefore, the objective of the current study was to assess the feasibility of nose-to-brain delivery of MEL LNCs for treatment of brain ischemia, and their characterization by suitable *in vitro*, *ex vivo*, and *in vivo* experiments. Till current date, there are no published reports on loading MEL in LNCs, therefore in the present preclinical study, we report the development and characterization of MEL-LNCs for nose-to-brain delivery, with pharmacodynamic proof of efficacy in cerebral ischemia/reperfusion (I/R) model in rats.

## Materials and methods

### Materials

MEL was purchased from skin actives scientific, USA. Solutol HS15, acetonitrile, and water (HPLC grade) were purchased from Sigma-Aldrich Co., Germany, Labrafil M1944 CS was kindly provided as a gift from Gattefosse Co., France. Epikuron 200 (Soya bean lecithin) was kindly provided as a gift from Cargill Co., Germany. Thiopental was purchased from Sigma-Tec Pharmaceutical Ind., Egypt.

### Preparation of MEL-LNCs

MEL-LNCs were prepared using the phase inversion temperature method **(**Heurtault et al., 2002; Lamprecht et al., 2004; Mohsen et al., 2020**)**. An amount of MEL ranging from 25 to 50 mg was dissolved in Labrafil (ranging in amount from 1 to 2.5 g), then mixed with Solutol HS15 (ranging in amount from 1 to 2.5 g), based on the amounts detailed in Table 1. Distilled water (3 mL) and soya bean lecithin (150 mg), in addition to the mixture were heated under magnetic stirring up to 85 °C for the formation of the w/o emulsion. The emulsion was then cooled to 55 °C, accompanied by phase inversion to o/w emulsion. This cycle was repeated twice before adding an amount of distilled water (to reach a final weight of 10 g) at 4 °C. The LNCs dispersion was then stirred for 10 min before further analysis. The final weight of the formulation components (oil, Solutol, water, and lecithin) was set to 10 g, excluding the weight of MEL.

**Table 1.** Particle size, polydispersity index, and zeta potential values of MEL-LNCs prepared according to the D-optimal mixture design.

Formula code	*X*_A_	*X*_B_	*X*_C_	Mean particle size (nm) ± SD	PDI	Zeta potential (mV) ± SD
LNC1	25	10	50	55.45 ± 3.46	0.18 ± 0.03	–0.60 ± 0.43
LNC2	10	10	35.23	33.23 ± 0.99	0.17 ± 0.07	–8.01 ± 0.08
LNC3	17.52	40	34.40	51.26 ± 58.32	0.23 ± 0.06	–6.90 ± 0.26
LNC4	10	40	35.63	18.72 ± 0.22	0.25 ± 0.01	–8.07 ± 0.52
LNC5	25	10	20	101.90 ± 1.45	0.24 ± 0.03	–6.60 ± 0.21
LNC6	10	10	20	35.50 ± 0.34	0.38 ± 0.00	–8.18 ± 0.10
LNC7	21.29	25	20	56.82 ± 0.42	0.10 ± 0.01	–5.50 ± 0.56
LNC8	10	40	20	23.05 ± 0.09	0.40 ± 0.02	–3.52 ± 0.11
LNC9	25	40	20	32.56 ± 0.07	0.53 ± 0.00	–3.06 ± 0.37
LNC10	20	10	35	58.06 ± 1.55	0.35 ± 0.04	–4.20 ± 0.37
LNC11	25	40	35	24.49 ± 0.02	0.21 ± 0.01	–4.66 ± 0.28
LNC12	17.66	25.80	50	55.27 ± 0.26	0.61 ± 0.04	–4.07 ± 0.83
LNC13 (LNC9′)	25	40	20	34.62 ± 0.80	0.55 ± 0.01	–4.58 ± 0.56
LNC14	25	40	50	29.38 ± 2.02	0.42 ± 0.04	–3.95 ± 0.32
LNC15	10	40	50	20.88 ± 0.61	0.33 ± 0.01	–1.17 ± 0.07
LNC16	10	25.67	20	19.74 ± 0.38	0.13 ± 0.00	–4.87 ± 0.20
LNC17	10	25.48	50	78.89 ± 3.33	0.25 ± 0.03	–6.09 ± 0.28
LNC18 (LNC15′)	10	40	50	18.26 ± 0.06	0.22 ± 0.00	–1.20 ± 0.30
LNC19 (LNC8′)	10	40	20	19.60 ± 0.09	0.29 ± 0.00	–4.52 ± 0.12
LNC20	16.94	10	50	65.53 ± 0.58	0.28 ± 0.00	–3.89 ± 0.12
LNC21	25	25	49.06	64.53 ± 0.28	0.12 ± 0.00	–3.32 ± 1.54
LNC22 (LNC6′)	10	10	20	30.66 ± 0.52	0.32 ± 0.00	–6.98 ± 0.12
LNC23	10	10	50	51.22 ± 0.72	0.45 ± 0.01	–6.84 ± 0.05
LNC24 (LNC5′)	25	10	20	109.80 ± 1.85	0.19 ± 0.01	–6.43 ± 2.60
LNC25 (LNC23′)	10	10	50	51.30 ± 0.73	0.42 ± 0.00	–6.90 ± 1.35
LNC26	18.06	10	20	74.54 ± 0.56	0.23 ± 0.02	–8.73 ± 0.34
LNC27 (LNC14′)	25	40	50	23.68 ± 5.33	0.28 ± 0.05	–1.20 ± 0.26
LNC28 (LNC1′)	25	10	50	45.97 ± 3.66	0.14 ± 0.01	–1.26 ± 0.58
LNC29	16.35	23.08	31.25	52.90 ± 17.98	0.23 ± 0.01	–2.72 ± 0.58
LNC30	25	23.88	34.36	51.24 ± 0.52	0.62 ± 0.00	–5.25 ± 0.24

*X*_A_: Labrafil concentration (wt/wt%); *X*_B_: Solutol concentration (wt/wt%); X_C_: Melatonin amount (mg). The gray-shaded formulations represent the 8 replicates (same concentration of oil/Solutol/drug) of other LNCs.

Design Expert 7.0 software (Stat-Ease, Inc.) was used to build D-optimal mixture design for studying the effect of three independent variables (concentration/amount of ternary blends); *X*_A_ (oil), *X*_B_ (Solutol HS 15), and *X*c (MEL) on the particle size of MEL-LNCs, as shown in Table 1. The bounds were chosen in an acceptable value range **(**Nguyen et al., 2014**)**, which in our case were reported to be 10%–40% for Solutol, 10%–25% for oil, 35%–80% for water, and 1.5% for lecithin **(**Mohsen et al., 2020**)**.

### Characterization of MEL-LNCs

#### Particle size, polydispersity index, and zeta potential measurements

Measurement of the particle size, polydispersity index (PDI), and zeta potential of MEL-LNCs was made using Zetasizer Nano (model ZS3600, Malvern, UK) **(**Ismail et al., 2020; Mohsen et al., 2020**)**.

### Measurement of viscosity

The viscosity of the selected MEL-LNCs was determined using Anton Paar rheometer connected to spindle no PP25. Measurements were made at room temperature at constant shear rate 7.34 s^−1^(60 rpm).

### *In vitro* drug release

Drug release was performed on the selected MEL-LNCs formulations using membrane diffusion technique, as previously described elsewhere **(**Mouez et al., 2016; Nasr, 2016**)**. One milliliter of the selected MEL-LNCs formulations (containing 5 mg MEL) was placed in glass cylinder fitted with the cellulose membrane (12,000–14,000 M.wt cut off), clamped at one end to the shaft of the dissolution apparatus (Pharma Test, Type PTW, Germany). The dissolution medium was 95 mL phosphate buffer pH 7.4 **(**Mao et al., 2004**)** as dissolution medium, ensuring sink condition for MEL at 37 °C. The cylinders were rotated at 100 rpm, and aliquots of the medium (1 mL) were taken at definite time intervals, and replaced by fresh dissolution medium. The amount of released MEL was quantified by UV spectroscopy (model 1800, Shimadzu, Japan) at *λ*_max_ 278 nm against blank formulation not containing MEL to account for any possible interference from the formulation components.

### Storage stability study

After three months, stability of the selected MEL-LNCs stored at 4 °C was evaluated by re-measuring the particle size, PDI, and zeta potential of the formulations **(**Brum et al., 2017; Mohsen et al., 2020**)**. In addition, MEL content in LNCs was re-assessed by dissolving an aliquot of LNCs in methanol, and quantification of MEL using high-performance liquid chromatography (HPLC) **(**Martins et al., 2017).

### Morphology of the selected MEL-LNCs formulation using transmission electron microscopy

Fifty microliters of diluted sample of the optimized formulation was adsorbed to a carbon-coated grid for 2 min and negatively stained using 50 µL of 2% uranyl acetate aqueous solution. The excess solution was removed from the grid, followed by transmission electron microscopy (TEM) examination (JEM-100 S, Japan).

### *Ex vivo* permeation of the selected MEL-LNCs formulation

The *ex vivo* animal experiment was approved by the research ethics committee of the Faculty of Pharmacy, Ain Shams University (Approval number REC-ASU56). All methods were performed in accordance with the relevant guidelines and regulations, and all experiments were performed in accordance with the ARRIVE guideline.

Isolated sheep nasal mucosa was used as an *ex vivo* model to monitor the nasal permeation of MEL-LNCs, since it is histologically identical to the human mucosa **(**Shaw et al., 2000**)**. The mucosa was collected from a local slaughter house and carefully cleaned from the adhered tissues under cold running water. The mucosa was placed in a Franz-type diffusion apparatus (Variomag Telesystem, Germany), and the permeation experiment was conducted as described elsewhere **(**Mouez et al., 2016; Nasr, 2016**)**. The receptor medium was 7.5 mL phosphate buffer (pH= 7.4) of temperature 37 ± 0.2 °C, the diffusion area of the cells was 1.77 cm^2^, and the amount of utilized formulation was 100 µL. Samples were taken at specified time intervals (5, 15, 30, 45, 60, 90, 120, 180, and 240 min), and analyzed using HPLC **(**Martins et al., 2017).

### *In vivo* study on cerebral/ischemia reperfusion model

Male Wistar rats, aged 10 weeks and weighing 250 ± 20 g, were housed 2 per cage at 25 °C, humidity 60%, and a 12/12 h dark/light cycle. Animals were supplied with standard diet chow and tap water, and were acclimatized for one week before the start of the experimental work. All procedures related to animal manipulation and treatments were approved by the research ethics committee of the Faculty of Pharmacy, Ain Shams University (Approval number REC-ASU56). All methods were performed in accordance with the relevant guidelines and regulations, and all experiments were performed in accordance with the ARRIVE guideline.

I/R insult was induced in rats according to previously reported methods **(**Collino et al., 2006; Awad, 2011**)**. After anesthesia with thiopental sodium (30–40 mg/kg) administered by intraperitoneal injection, ischemia was induced by bilateral ligation of the common carotid arteries using non-traumatic mini-clips. Sixty minutes later, both carotid arteries were declamped to assist reperfusion either for 24 h or 5 days, and the incisions were sutured.

Rats were divided into two sets, each with four groups (*n* = 8 per group). In both sets, the first group served as the sham-operated group and the second one was the I/R group. Groups 3 and 4 rats were treated with MEL in saline, and MEL-LNCs (LNC-15) respectively by intranasal administration (50 µL in each nostril) by means of micropipette linked to polyethylene tube (0.1 mm) inserted 5 mm deep in each nostril **(**Kumar et al., 2008; Haque et al., 2012; Xiao et al., 2016**)** at 0 h, 2 h, and 6 h following the termination of ischemic period **(**Kavakli et al., 2004; Awad, 2011; Hu et al., 2016**)**.

One day (24 h) after ischemic insult, animals in the first set were sacrificed by an overdose of thiopental sodium (50–60 mg/kg) administered by intraperitoneal injection, and the brains were harvested and the two hippocampi in both hemispheres were dissected on ice-cold plates and stored at –80 °C for the biochemical and western blot investigations according to the manufacturer’s procedures **(**Abd El-Aal et al., 2017**)**. Animals in the second set were sacrificed 5 days following ischemic period and used for histopathological estimation after hematoxylin and eosin (H&E) staining.

#### Histopathological assessment

The number of intact viable neurons in CA1 region of stained hippocampal tissues was taken as an indication of hippocampal damage. Neurons displaying distinctive nuclei and nucleolus surrounded by intact cell membrane were counted using image analyzer software (Leica Qwin 500, United Kingdom). The mean number of survived neurons in CA1 subfield was determined in three successive hippocampal sections/rat (8 rats/group).

#### Assessment of oxidative stress

Hippocampal lipid peroxide expressed as malondialdehyde (MDA) (Cat. No. MD 2529) and antioxidant molecules recognized as superoxide dismutase (SOD) (Cat. No. SD 2521) and glutathione (GSH) (Cat. No. GR 2511) were quantified using commercial kits purchased from Bio-diagnostic, Giza, Egypt according to the manufacturer’s procedures.

#### Assessment of pro-inflammatory cytokines and apoptotic biomarkers

After processing following the designated manufacturers’ protocols, the hippocampal supernatants were used to quantify myeloperoxidase (MPO) (Cat. No. ab105136, Abcam, UK), tumor necrosis factor-α (TNF-α) (Cat. No. ELR-TNFa, Ray Biotech, Norcross, USA), nitric oxide (NO*_X_*) (Cat. No. K023-H1, Arbor Assays, USA), and Caspase-3 activity (Cat. No. K106-25, R&D system, USA).

#### Western blot analysis

Hippocampal cytosolic, mitochondrial, and nuclear lysate fractions were prepared for western blotting as previously detailed elsewhere **(**Abd El-Aal et al., 2017; El-Gazar et al., 2019**)**. The amount of protein in each sample was estimated (Cat. No. TP 2020, Bio-diagnostic, Egypt), and equal amount of protein (50 µg/sample) was resolved by precast 10% SDS-PAGE gel, then transferred to 0.45-µm nitrocellulose membrane (Cat. No. 88018, Thermo Fisher, USA). The membranes were blocked by 5% wt/vol skimmed dry milk for 60 min with gentle shaking, and the proteins of interest were incubated/probed overnight at 4 °C with the primary antibodies (Abcam, UK) against Bax (1:100, Cat. No. ab53154), Bcl-2 (1:200, Cat. No. ab59348), cytochrome c (1:200, Cat. No. ab13575), Hemoxygenase-1 (HO-1, 1:200, Cat. No. ab13243), inducible nitric oxide synthase (iNOS, 1:200, Cat. No. ab15323), Nrf-2 (1:200, Cat. No. ab137550), Bcl-2 (1:200, Cat. No. ab59348), phosphorylated nuclear factor kappa B (*p*-NF-κB p65, 1:1000, Cat. No. ab86299), and β-actin (1:1000, Cat. No. ab8227). Twenty-four hours later, the membrane was washed three times in TBS/tween 20 and incubated with horseradish peroxidase-labeled secondary antibodies (1:1000, Cat. No. 7076S, Cell signaling technology, USA) for 60 min at room temperature. Expressed proteins were detected using enhanced chemiluminescence substrate (BioRad, USA). Densitometric analysis of expressed signals was performed via ChemiDoc imaging system using Lab software version 5.1 (BioRad, USA).

### Statistical analysis

*In vitro* experimental data were expressed as mean ± SD (*n* = 3). Results were compared using one-way analysis of variance (ANOVA) followed by Tukey Kramer post-test at a level of significance *p* ≤ .05 using Graphpad Instat software. For the *in vivo* study, data were expressed as mean (*n* = 8) ± SEM and statistical comparisons were performed using one-way analysis of variances followed by Tukey’s post hoc test. The figures were generated using Microsoft Excel 2007 and Graphpad prism software version 8.1.

## Results and discussion

### Preparation of MEL-LNCs

The phase inversion temperature method (PIT) was chosen for the preparation of LNCs due to its several reported advantages such as simplicity of the procedure, low cost, and reproducible particle size **(**Heurtault et al., 2002; Huynh et al., 2009**)**. The Solutol HS was the most commonly used nonionic surfactant for the preparation of LNCs using the PIT method **(**Anton et al., 2008**)** while the lipophilic surfactant (Soya bean lecithin) was used in small proportions to increase LNCs stability by creating a ‘framework’ in the shell **(**Minkov et al., 2005; Vonarbourg et al., 2005**)**. Labrafil (oleoyl polyoxyl-6 glycerides NF) was used as the oil phase based on preliminary experiments. MEL was successfully loaded in LNCs, as shown by the comparable UV–vis spectrum of the methanolic solution of MEL to that of MEL-loaded LNCs (using plain LNCs as blank) (Supplementary 1).

### Particle size, polydispersity index, and zeta potential measurement

As shown in Table 1, all MEL-LNCs formulations showed mean particle size up to 110 nm (ranging from 18.26 to 109.8 nm), which is a highly recommended size for brain delivery **(**Danaei et al., 2018; Rizvi & Saleh 2018**)**, especially through the intranasal route **(**Clementino et al., 2016; Mohsen et al., 2020; Sabir et al., 2020**)**. Formulations (LNC4, LNC15, LNC16, LNC18, and LNC19) showed the smallest particle size in the range of 20 nm as they had the highest concentration of Solutol.

D-optimal factorial design was built up to study the effect of three factors on the particle size of MEL-LNCs, which are the concentration of labrafil oil, Solutol HS15, and the amount of MEL. As shown in Table 1, it is clear that Solutol concentration (*X*_B_) was found to have a significant effect on the particle size of MEL-LNCs, in which increasing the Solutol concentration from 10% to 40% at both 25% labrafil concentration (LNC1/LNC14), (LNC5/LNC9), (LNC24/LNC13), (LNC28/LNC27) and 10% labrafil concentration (LNC6/LNC8), (LNC23/LNC15), (LNC25/LNC18), and (LNC22/LNC19) significantly decreased the particle size of nanocapsules (*p* < .05). These results were consistent with other reports, and could be ascribed to the fact that Solutol molecules primarily reside at the oil–water interface, hence lowering the interfacial tension and contributing to the dispersion of oil, which leads to small particle size **(**Lamprecht et al., 2002; Huynh et al.,^2009^; Zhai et al., 2018; Ismail et al., 2020**)**. The oil concentration (*X*_A_) had no significant effect on the particle size of MEL-LNCs (*p* > 0.05), which complied with the results of other authors **(**Khosa et al., 2018**)**. Finally, according to the generated model, increasing the drug amount throughout the range of 20–50 mg led to an overall increase in the particle size of the nanocapsules (*p* < 0.05), with some exceptions, on the contrary to what was reported in other studies, which stated that preparing LNCs using the same technique confirmed that the particle size was mainly affected by the surfactant concentration **(**Brum et al., 2017; Mohsen et al., 2020**)**. The model obtained is represented by the following equation:

$$\begin{array}{l} \text{Particle size }=\;+\text{64}.\text{81 }-\text{6}.\text{98}A\;-\text{711}.\text{51}B\;+\text{ 427}.\text{76}C\;-\text{6}.0\text{7}\mathrm{AB}\;-\text{1}0.\text{55}\mathrm{AC}\;+\text{3}.\text{3}0\mathrm{BC}\;-\text{29}.\text{97}A^{\text{2}}-\\ \text{5}.\text{93}B^{\text{2}}+\text{14}.0\text{8}C^{\text{2}}+\text{8}.\text{51}\mathrm{ABC}\;-\text{24}.\text{13}A^{\text{2}}B\;-\text{ 5}.0\text{9}A^{\text{2}}C\;+\text{39}.\text{2}0AB^{\text{2}}-\text{ 4}.\text{56}AC^{\text{2}}-\text{16}.\text{67}B^{\text{2}}C\;-\text{2}.0\text{1}BC^{\text{2}}-\\ \text{16}.\text{35}A^{\text{3}}+\text{719}.\text{91}B^{\text{3}}-\text{ 411}.\text{51}C^{\text{3}} \end{array}$$

The statistical validation displayed extreme significance for the proposed model (*p* < 0.0001). The model’s *R*^2^ was considered adequately high 0.985 with an adjusted *R*^2^ of 0.958, showing a good correlation with the data. The obtained signal-to-noise ratio of the obtained model was 21.66, which is greater than 4, indicating the suitability of the model to navigate the design space **(**Abd-Allah et al., 2020**)**. In addition, the Box–Cox power transformation suggested that no transformation is required for the suggested linear equation.

As also shown from the results, the PDI values of all LNCs mostly did not exceed 0.4, suggesting a homogeneous and monodisperse population for most of the formulations **(**Danaei et al., 2018; Zhai et al., 2018; Fadel et al., 2021**)**.

As also shown in Table 1, MEL-LNCs exhibited negative surface charges in the range of –0.60 to –8.73. Similar zeta potential values were obtained with other authors for LNCs **(**Coradini et al., 2014; Dos Santos et al., 2015**)**. The negative zeta potential values of the LNCs could be attributed to the components of the nanocapsules; namely the PEG dipoles in Solutol, and the presence of lecithin which is zwitterion surfactant exhibiting a negative charge at pH values higher than its isoelectric point (4.15) **(**Petelska & Figaszewski, 2002; Schuh et al., 2014**)**. Despite the low values of zeta potential of the prepared LNCs, it is expected that the PEG moieties on the surface of the LNCs would confer steric stabilization, similar to what was obtained by other authors **(**Mouzouvi et al., 2017**)**.

The MEL-LNCs formulations with high MEL loading of 50 mg (LNC1, LNC12, LNC14, LNC15, LNC17, LNC20, and LNC23) were selected for further characterization.

### Measurement of viscosity of the selected MEL-LNCs

The viscosity values of MEL-LNCs were found to be in the range of (1–1.74 cP), similar to what was obtained by other authors **(**Contri et al., 2013; Dos Santos et al., 2015**)**. The viscous nature of Solutol led to a slight yet significant increase in the viscosity of LNCs by increasing its concentration from 10% to 40% (*p* < .05) (LNC 15/17/23) and (LNC 1/14), while the change in oil concentration did not exhibit a specific trend on the viscosity of LNCs.

### *In vitro* drug release for the selected MEL-LNCs

**Figure 1.**
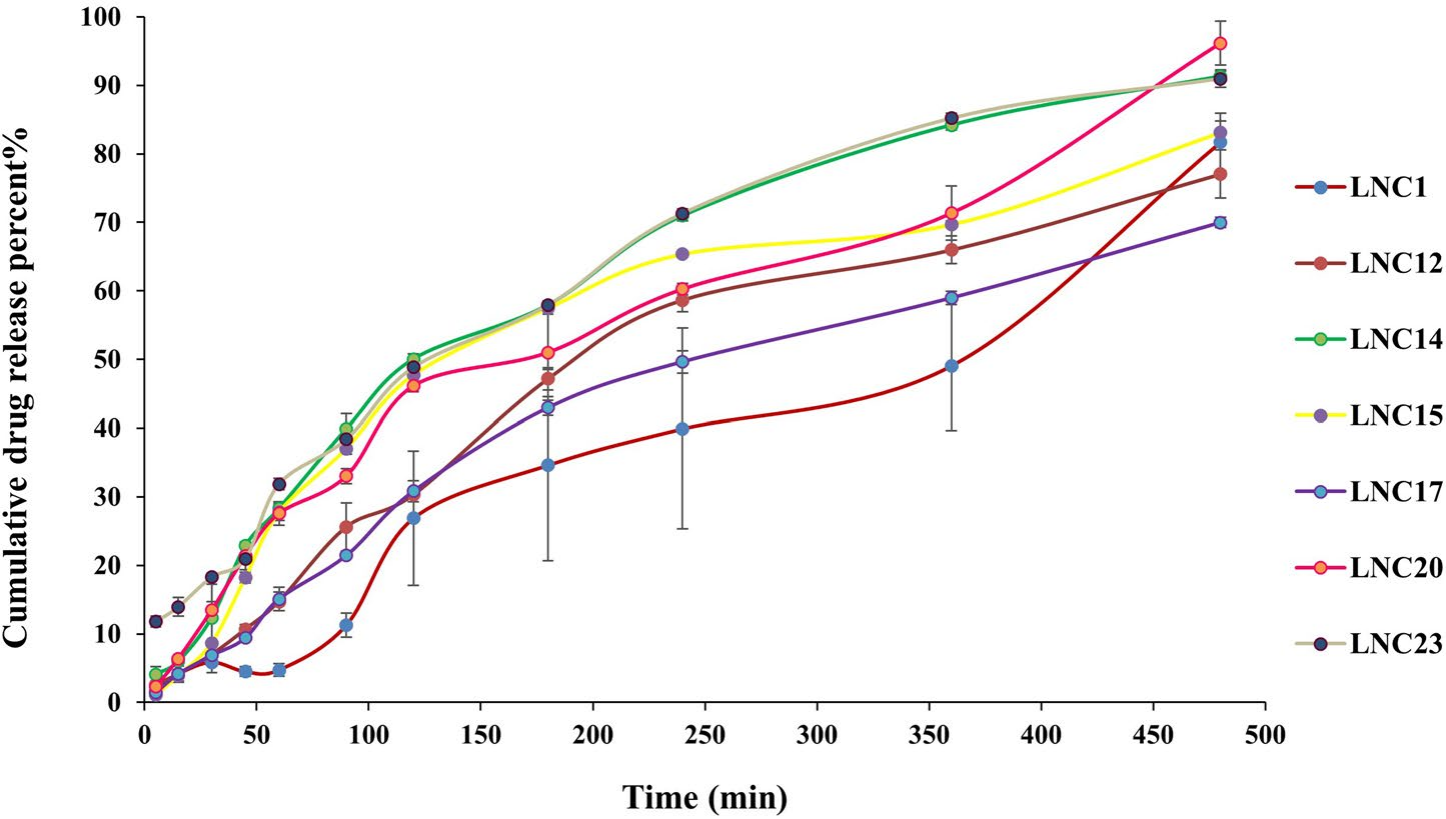
*In vitro* release profiles of MEL from the selected formulations of melatonin lipid nanocapsules (MEL-LNCs) in phosphate buffer (pH 7.4) at 37 °C (*n* = 3).

As shown in Figure 1, the cumulative release percent of MEL from the selected LNCs formulations ranged from 69.97 to 96.16%. LNCs formulations (except for LNC23) showed only a small initial burst effect followed by slow sustained release of the encapsulated drug, which concurred with the results of Lamprecht et al. (2002). The high burst release of LNC23 (11.78% after 5 min) may be attributed to the fact that it exhibits the lowest viscosity compared to the other formulations (*p* < 0.05) (1.04 cp for LNC23 compared to 1.43–1.74 cp for the other formulations). By further inspection of the cumulative percent released of MEL after 8 h, it could be inferred that the concentration of Solutol or oil did not exhibit a specific trend on the cumulative amount of the drug released after 8 h.

### Stability study

As could be inferred from the results in Supplementary 1, significant increase in the particle size and PDI was obvious in the formulations after storage (*p* < .05), with mostly insignificant changes in the zeta potential values of MEL-LNCs (*p* > .05). This suggests that steric stabilization by Solutol was not sufficient for most of the formulations (LNC 1/12/14/17/20/23), and the low zeta potential values **(**Mazzarino et al., 2010**)** actually led to aggregation of the LNCs on storage, only when Solutol was present in high concentration (40%) in conjunction with low concentration of oil (10%) in formulation LNC15, a slight increase in the particle size from 20.88 to 23.5 nm was evident, with non-significant change in PDI and zeta potential values. This implies that the composition of LNCs plays a role in dictating its stability properties. Worthy to note is that the loading of MEL was preserved in the LNCs with no significant change, and there was no evidence of degradation in the HPLC chromatograms, suggesting its stability in the formulation. Based on the aforementioned, LNC15 composed of 10% labrafil oil, 40% Solutol HS 15, and 50 mg MEL was selected for further characterization experiments, since it exhibited the best stability properties and smallest particle size among its tested LNCs counterparts.

### Morphology of the selected LNCs

**Figure 2.**
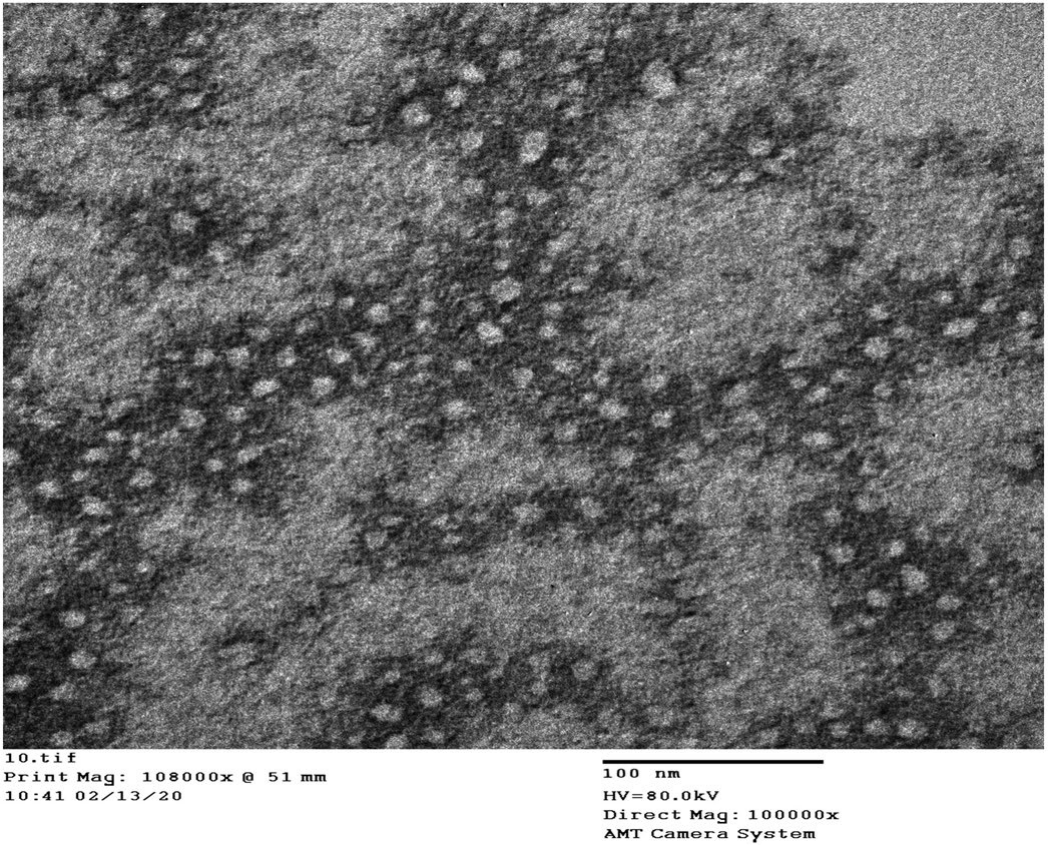
Negative stain electron micrograph of formula LNC15 at a magnification of ×100,000.

The TEM image in Figure 2 shows LNC15 morphology of sealed spherical structure with particle size matching that obtained using the Zetasizer. A similar morphology was also reported by Mohsen et al. (2020).

### *Ex vivo* permeation across sheep nasal mucosal

*Ex vivo* permeation data represented in Table 2 shows that LNCs were able to provide significantly higher MEL permeation across excised sheep nasal mucosa in comparison with its control solution after 6 h (*p* < .05), in which the permeation of MEL LNCs was 10.35-folds higher than drug solution. The superiority of the selected formulation (LNC15) compared to the solution form could be ascribed to its high content of the nonionic surfactant Solutol (40%), which induces reversible modifications on the structure of the nasal epithelial barrier by leaching of membrane proteins, opening of tight junctions **(**Brunner et al., 2021**)**. The permeability enhancing effect could be related to Solutol’s partitioning into the cellular membrane structure at physiological temperature. Solutol HS15 was capable of enhancing the nasal bioactivity of parathyroid hormone from 7.8% to 78% **(**Williams et al., 2018**)**. Furthermore, lecithin (phospholipid) also acts as an absorption enhancer across mucosal layers **(**Li et al., 2015; Ghadiri et al., 2019**)**. Moreover, the small size of the LNCs (20 nm) is expected to have significant contribution on the permeation across the nasal mucosa, as reported by other authors **(**Mohsen et al., 2020**)**.

**Table 2.** *Ex vivo* MEL-permeation data on LNC15 compared to its control.

Time (min)	MEL-LNC cumulative release %	MEL-solution cumulative release %
0	0 ± 0	0 ± 0
5	6.20 ± 12.69	0 ± 0
15	20.69 ± 7.27	0 ± 0
30	24.02 ± 5.76	0 ± 0
45	26.96 ± 3.57	0 ± 0
60	30.02 ± 1.77	0.19 ± 0.11
90	33.21 ± 0.17	0.39 ± 0.13
120	35.95 ± 0.10	0.67 ± 0.33
180	40.22 ± 3.34	1.73 ± 0.62
240	43.39 ± 5.48	3.46 ± 1.68
360	45.97 ± 8.75	4.44 ± 1.53

### *In vivo* studies on the effect of MEL-LNCs on cerebral/ischemia reperfusion

#### Effect of MEL-LNCs on I/R-triggered hippocampal architecture damage

**Figure 3.**
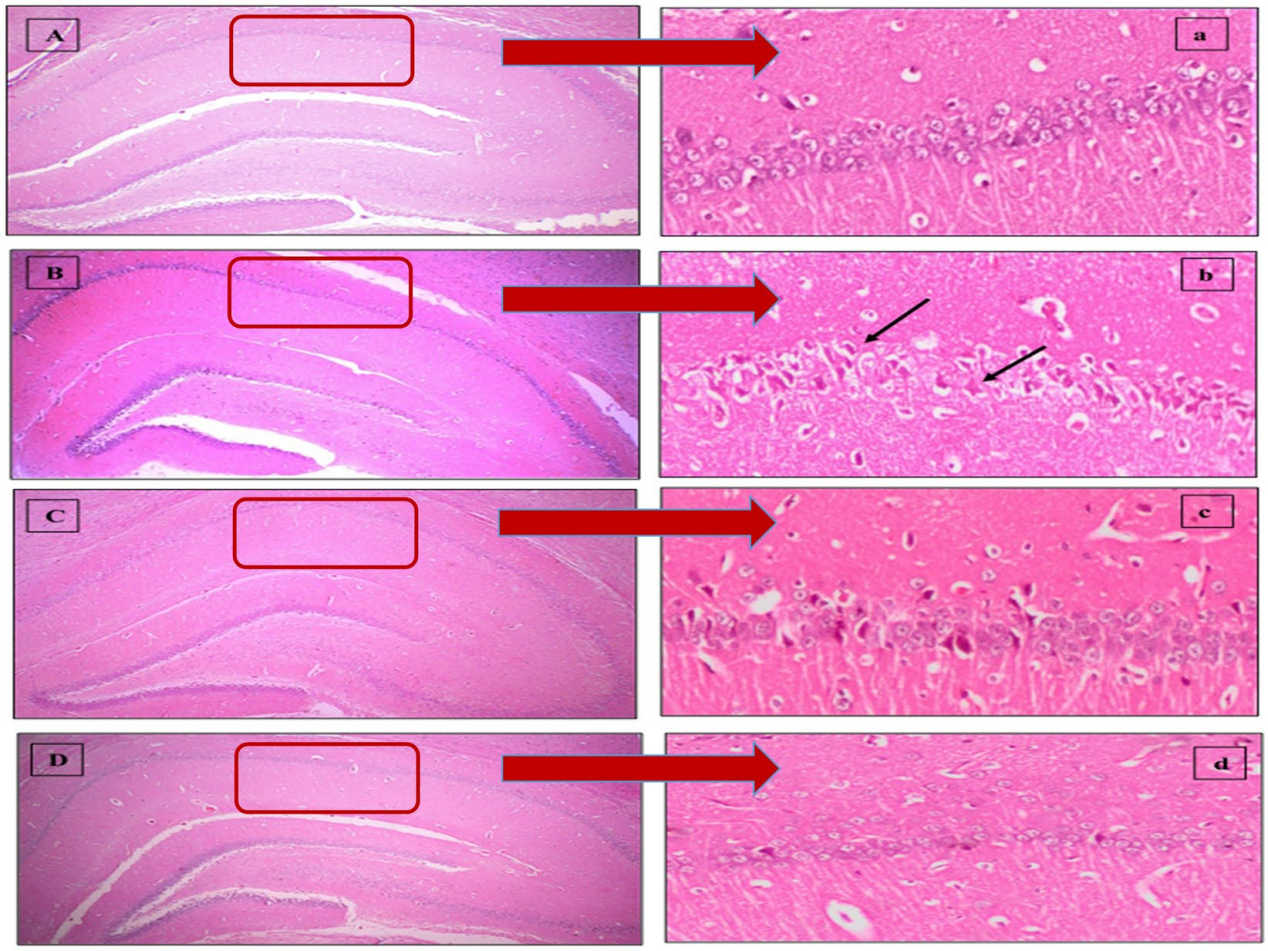
Descriptive photomicrographs (×400) of hematoxylin and eosin (H&E) staining (upper panel) revealing the neuroprotective impact of melatonin (MEL) on hippocampal CA1 region. (A,a) Sham, (B,b) Ischemia/reperfusion (I/R), (C,c) MEL solution-treated group, and (D,d) lipid nanocapsules LNC-treated group. The areas in the low field pictures (A,B,C,D) which are magnified as high power field pictures are denoted by red rectangles. Black arrows point at injured neurons.

**Figure 4.**
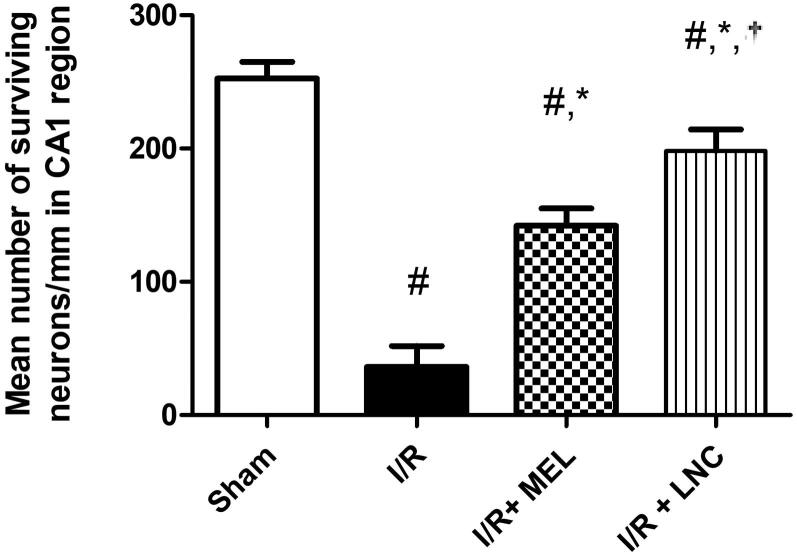
Mean number of surviving neurons in each group. Significance (*p* < .05) from sham group represented by (#), from ischemia/reperfusion (I/R) group represented by (*), and from melatonin (MEL solution-treated group) represented by (†).

As shown in Figure 3, photomicrographs (Figure 3(A,a)) obtained from sham rat sections displayed intact neurons with firm architecture in the hippocampal area. In contrast, I/R elicited selective and extensive damage in CA1 area 5 days following ischemic insult (Figure 3(B,b)); manifested as neurons with shrunken cytoplasm with pyknotic nuclei (black arrow). This was accompanied with decline in the number of viable neurons. Post-ischemic administration of MEL-LNCs (Figure 3(D,d)) opposed these aberrations with significant restoration of hippocampal neurons, which was found superior to that mediated by MEL solution (Figure 3(C,c)). The mean number of surviving neurons for each group is shown in Figure 4.

#### Effect of MEL-LNCs on oxidative stress, inflammation, and apoptotic biomarkers

Although restoration of blood flow is one of the therapeutic implements following ischemic insults, the generation of large amount of ROS that accompanies the ischemic insult results in cerebral damage by various mechanisms. The injurious impact spreads from direct oxidation of proteins, lipids, and DNA to the upregulation of transcriptional factors, such as NF-κB, which plays a critical role in inflammation. NF-κB aggravates ischemia via the activation of detrimental genes involved in the pathogenesis of hippocampal damage, viz NO, TNF-α, iNOS **(**Awad, 2011; Andrabi et al., 2015**)**.

**Figure 5.**
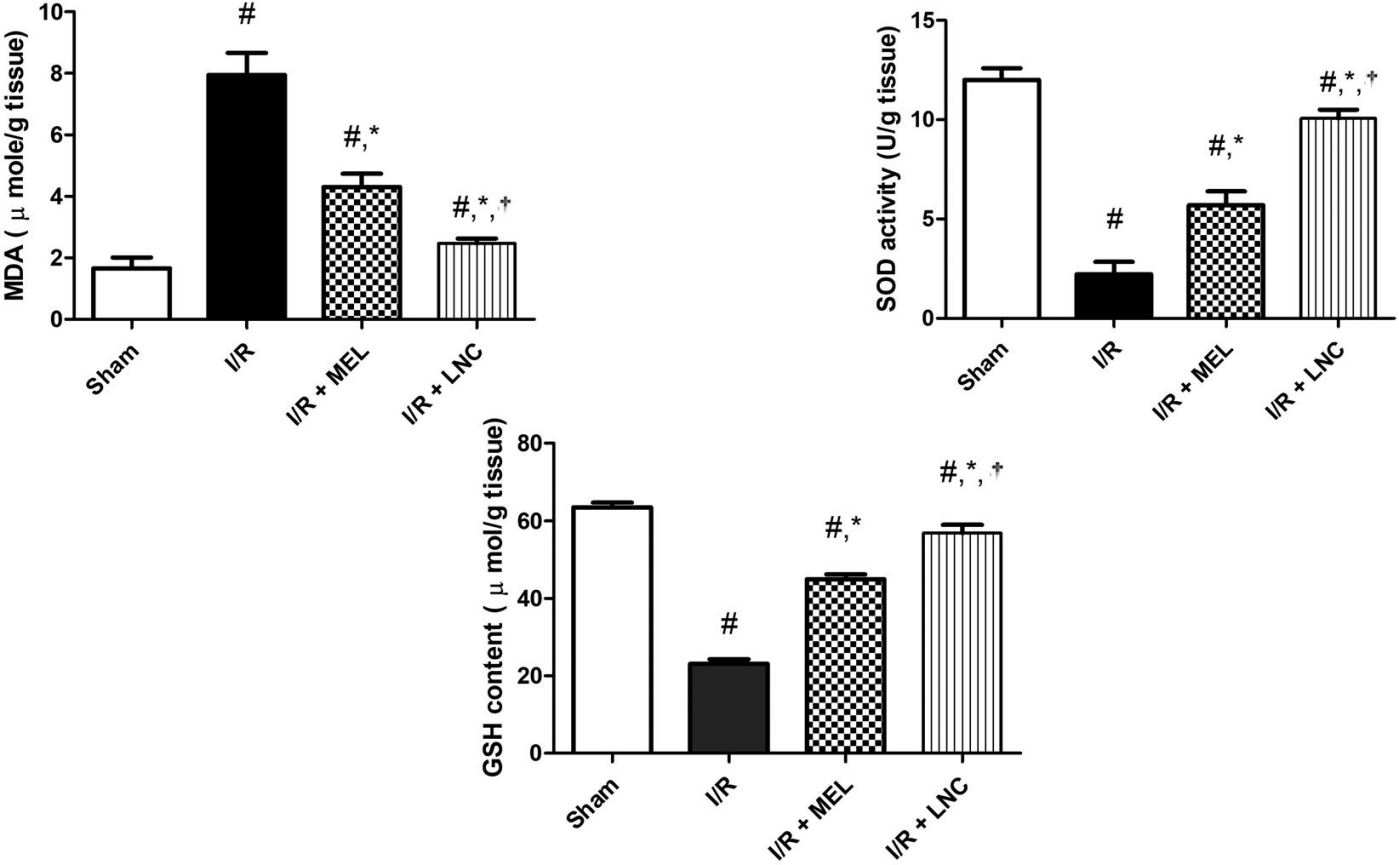
Oxidative stress biomarkers malondialdehyde (MDA), superoxide dismutase (SOD), and glutathione (GSH) levels for different groups. Significance (*p* < .05) from sham group represented by (#), from ischemia/reperfusion (I/R) group represented by (*), and from melatonin (MEL) solution-treated group represented by (†).

In agreement with the aforementioned, our study revealed that I/R provoked hippocampal oxidative stress (Figure 5**)**, manifested by the significant elevation of the activity of MDA (a familiar marker for lipid peroxidation) and decline of the cellular antioxidant defenses GSH and SOD, compared to sham group (*p* < .05). However, the intranasal administration of MEL-LNCs and MEL in saline at 0 h, 2 h, and 6 h after onset of ischemia halted hippocampal oxidative stress, manifested by significant reduction of MDA with concomitant increase in GSH and SOD levels (*p* < .05), with MEL-LNCs exhibiting significantly better antioxidant performance than MEL solution (*p* < .05).

**Figure 6.**
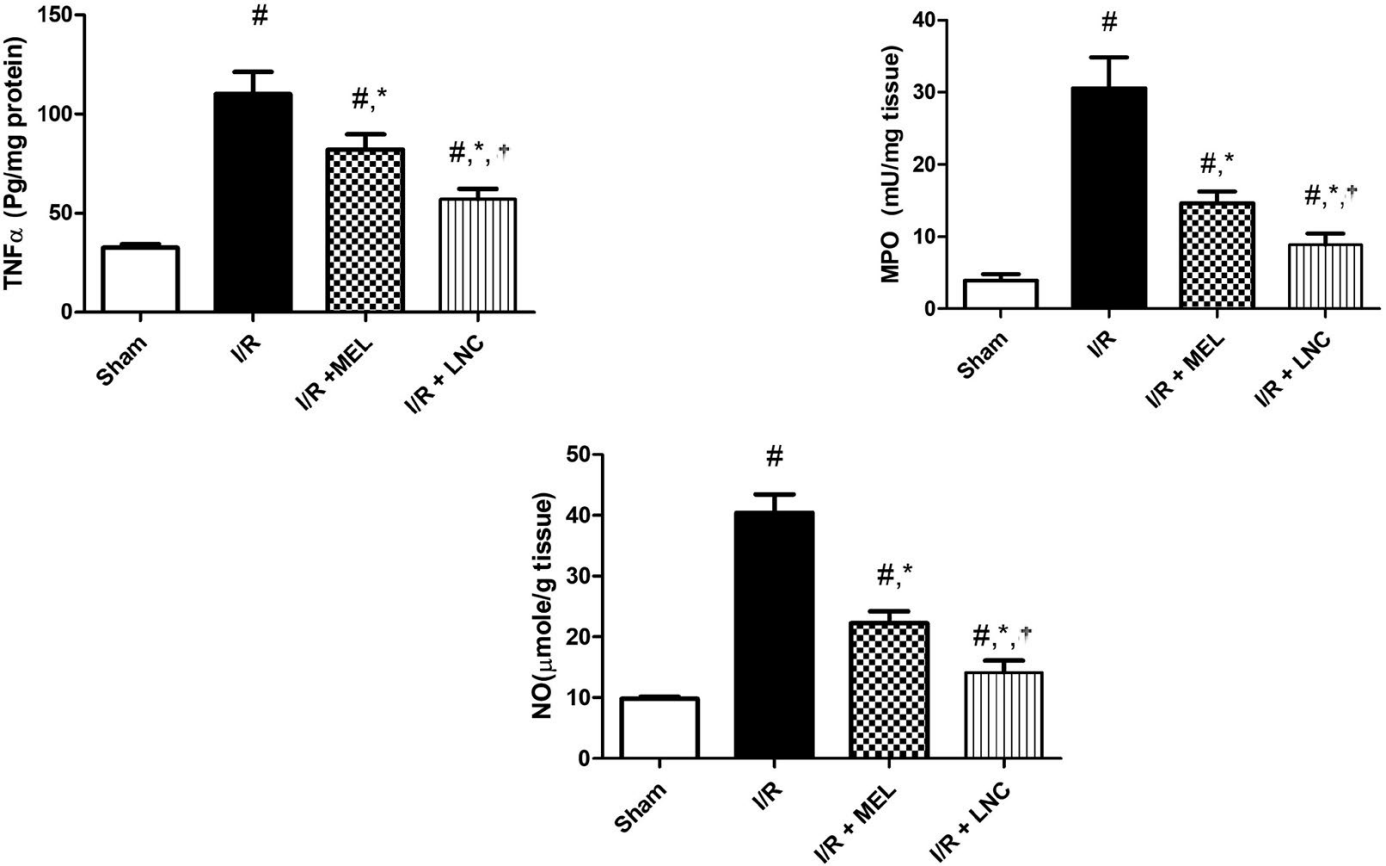
Inflammatory biomarkers tumor necrosis factor-α (TNF-α), myeloperoxidase (MPO), and nitric oxide (NO) levels for different groups. Significance (*p* < .05) from sham group represented by (#), from ischemia/reperfusion (I/R) group represented by (*), and from melatonin (MEL) solution-treated group represented by (†).

As also depicted in Figure 6, I/R resulted in inflammatory spike, evidenced by increased hippocampal content of TNF-α, MPO, and NO activity when compared to sham group. I/R also mediated an unpredictable downregulation of nuclear Nrf-2 and cytosolic HO-1; a finding that coincides with the observed upregulation of pro-inflammatory transcription factor NF-κB, which competes with Nrf2 for binding with nuclear motif CREB-binding protein, hence limiting its availability and hindering the Nrf-2 transcription and antioxidant strength **(**Tonelli et al., 2018**)**. Intranasal treatment with either MEL solution or MEL-LNCs resulted in upregulation of Nrf-2 and its target HO-1, significant reduction of hippocampal inflammation by curbing the expression of TNF-α, iNOS, NO and NF-κB p65, along with MPO activity (*p* < .05). Concurring with the antioxidant biomarkers results, MEL-LNCs exhibited significantly better anti-inflammatory performance than MEL solution (*p* < .05).

**Figure 7.**
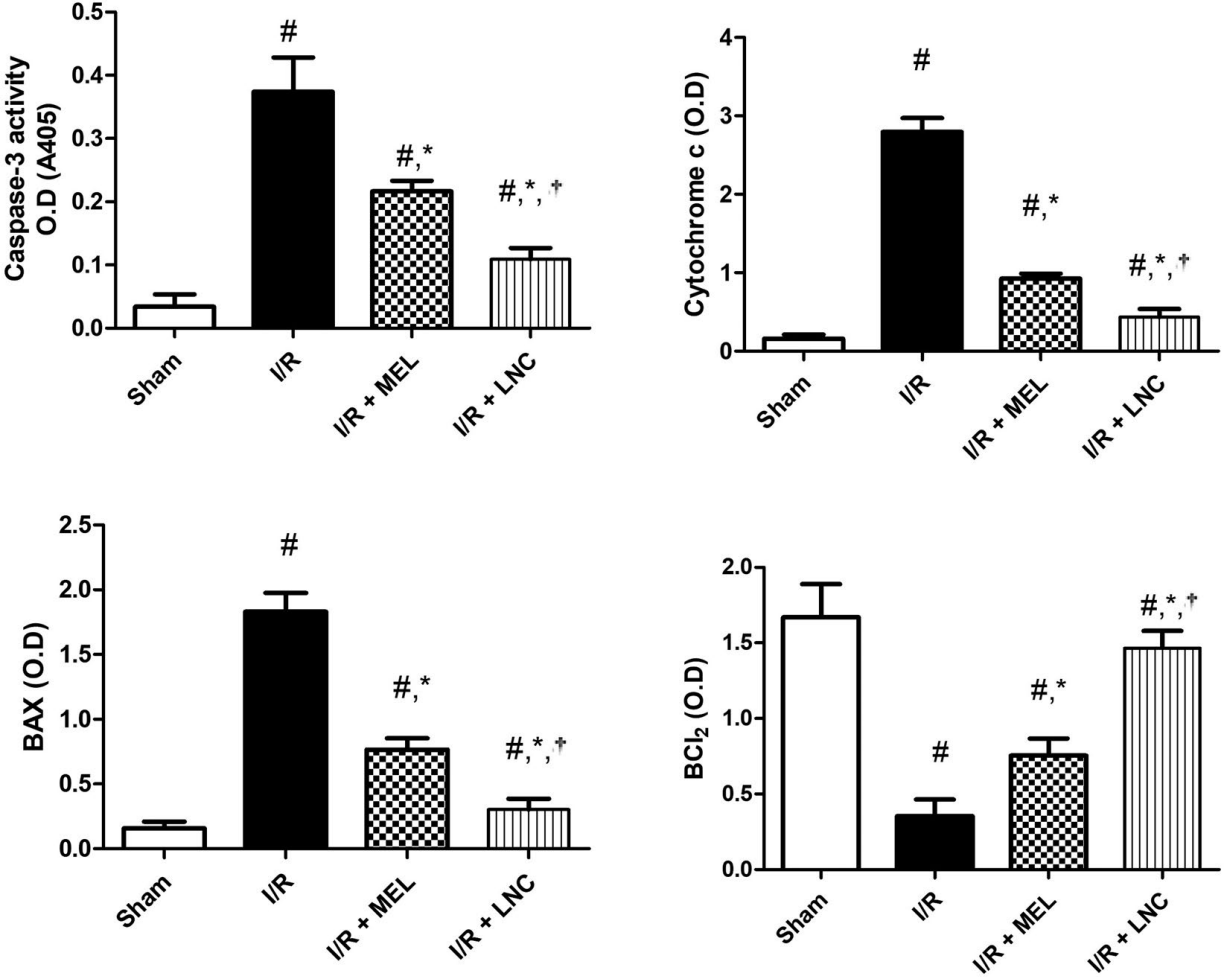
Apoptotic markers (caspase-3, cytochrome c, Bax, Bcl-2) for different groups. Significance (*p* < .05) from sham group represented by (#), from ischemia/reperfusion (I/R) group represented by (*), and from melatonin (MEL) solution-treated group represented by (†). Samples containing equal amount of protein lysates (50 µg per lane) were used for the analysis after processing, and overnight incubation at 4 °C with the antibodies. After 24 h, samples were incubated with horseradish peroxidase-conjugated secondary antibodies for 1 h at room temperature and protein blots were detected using enhanced chemiluminescence substrate.

Finally, I/R ischemic insult triggered hippocampal apoptosis, resulting in significant induction in the apoptotic markers (cytochrome *c*, Bax, and caspase-3), with significant downregulation of the pro-surviving anti-apoptotic B cell lymphoma-2 protein (Bcl-2) from sham group (*p* < .05) (Figure 7**)**. However, treatment with MEL-LNCs or MEL solution significantly averted all these consequences in favor of cell survival (*p* < .05), with the former being significantly superior to the latter (*p* < .05).

Combining all aforementioned data together, it could be inferred that the MEL-mediated attenuation of ROS generation can be attributed to the enhancement of Nrf-2/HO-1 pathway, similar to what was obtained upon MEL administration in animal models of diabetic neuropathy **(**Pourhanifeh et al., 2020**)**, subarachnoid hemorrhage-induced brain damage **(**Guo et al., 2018**),** and restraint-induced testicular damage **(**Guo et al., 2017; Sukhorum et al., 2020**)**. Besides reviving the I/R-mediated disruption of Nrf-2/HO-1 as a protective pathway, MEL extended its impact to entail inflammatory signaling pathway as well, in which as revealed by the current study, MEL suppressed I/R-mediated upregulation of hippocampal inflammation manifested by attenuating the activity of NO, TNF-α, and iNOS along with MPO (a reliable marker of neutrophils recruitment), in line with previous reports **(**de Oliveira Ferreira et al., 2016**)**. These anti-inflammatory impacts of MEL could be ascribed to the noted down regulation of transcription factor NF-κB p65, which was demonstrated to upregulate the transcription of the measured genes, iNOs and TNF-α. The latter is recognized to trigger neutrophil infiltration viz increasing MPO activity proved herein **(**Bao et al., 2004**)**. This can be further explained by the observed upregulation of pro-apoptotic proteins/enzyme, cytochrome *c*, Bax, and caspase-3 along with the lessening of Bcl-2 levels. The present data affirmed the neuroprotective/anti-apoptotic character of MEL and MEL lipid nanocapsules, verified herein by abating hippocampal death, apoptotic markers, with concomitant amplification of pro-surviving protein Bcl-2. The superiority of MEL-LNCs to MEL solution may imply more interaction of the former with MEL receptors (either MT1, MT2, or both), which are prominent all through the brain and thus, serve as a potent therapeutic target in the treatment of stroke **(**Acuña-Castroviejo et al., 2014**;** Wongprayoon and Govitrapong, 2021**)**. It could also be attributed to the better diffusion of LNCs across the sheep nasal mucosa, concurring with the *ex vivo* permeation results, which consequently leads to better uptake by epithelial cells, or neurons present in the nasal cavity, hence reaching the brain in a better manner **(**de Oliveira Junior et al., 2019**)**. In a study comparing the blood and brain pharmacokinetics of nimodipine following its intranasal administration in lipid nanocapsular form and intravenous solution form, the authors concluded that the intranasal administration of nimodipine lipid nanocapsules led to significantly higher amounts of the drug in the brain, with higher brain/blood ratios, and elucidated that the primary mechanism of nasal permeation was their ability to permeate across the blood brain barrier **(**Mohsen et al., 2020**)**. Therefore, it can be hypothesized that differences in vehicle characteristics between MEL and MEL-LNCs in our study affected their efficacy after intranasal administration, and this suggests that conduction of futuristic pharmacokinetic experimentation is required to verify this hypothesis.

Therefore, MEL lipid nanocapsules were delineated as promising neuroprotective therapy for ischemic injuries, with marked suppression of oxidative stress, inflammation, and apoptosis. However, among the limitations of the current study is the lack of pharmacokinetic data (in both the blood and brain) for MEL in rats owing to the complexity of MEL analysis in biological tissues, which could have provided a solid correlation with the obtained pharmacodynamic results, and could have facilitated the clinical translation of the results. Although its absence does not affect the conclusion of the study, futuristic studies would benefit from continuation of this work from a pharmacokinetic perspective. Another limitation is that the scalability of lipidic nanocapsules has not been reported in the literature, and this aspect has to be verified before clinical translation into marketed product is possible. Therefore, futuristic work would also benefit from a scaling-up study for lipidic nanocapsules.

## Conclusions

In this current work, MEL-loaded lipidic nanocapsules with favorable physicochemical properties were successfully prepared, with demonstrated high *ex vivo* nasal permeation, coinciding with promising neuroprotective actions *in vivo*, which suggests their promising therapeutic potential in mitigating the consequences of ischemic stroke. Future work will include further confirmation of the therapeutic potential with pharmacokinetic assessment of MEL concentration in the brain, blood, and non-target organs following intranasal administration of MEL nanocapsules, in addition to the elucidation of their biosafety.

## Supplementary Material

Supplemental Material
