# Supplementary material for: Nose to brain delivery of melatonin lipidic nanocapsules as a promising post-ischemic neuroprotective therapeutic modality
Source: Drug Deliv. 2022 Jul 27;29(1):2469–80. doi: 10.1080/10717544.2022.2104405 (PMC9341381; doi:10.1080/10717544.2022.2104405)
Supplement: Supplemental Material [file IDRD_A_2104405_SM7264.docx]

**UV-Vis spectra of melatonin and melatonin LNCs showing the characteristic λmax of melatonin at 278 nm**

**
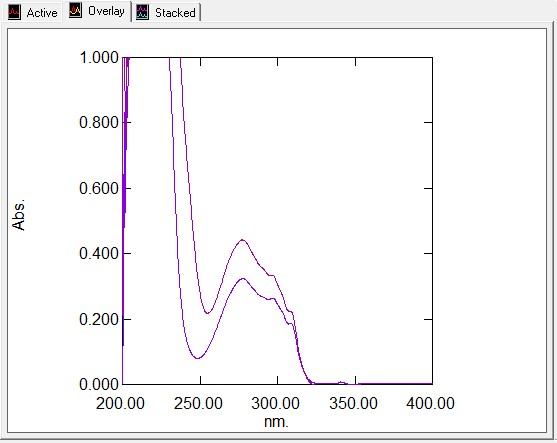
**

**Effect of storage on the particle size, PDI and zeta potential of the selected MEL-LNCs**

| **Formula code** | **Mean Vesicles particle size**  **(nm)±SD** | | **Mean Vesicle**  **PDI** | | **Mean vesicles**  **Zeta potential**  **(mV)±SD** | |
| --- | --- | --- | --- | --- | --- | --- |
|  | **Before storage** | **After storage** | **Before storage** | **After storage** | **Before storage** | **After storage** |
| **LNC1** | 55.45  ±3.46 | 188.7  ±2.08 | 0.18  ±0.03 | 0.48  ±0.33 | -0.60  ±0.43 | -0.48  ±0.40 |
| **LNC12** | 55.27  ±0.26 | 83.79  ±0.85 | 0.61  ±0.04 | 0.83  ±0.006 | -4.07  ±0.83 | -1.17  ±0.80 |
| **LNC14** | 29.38  ±2.02 | 50.08  ±0.30 | 0.42  ±0.04 | 0.59  ±0.01 | -3.95  ±0.32 | -4.81  ±1.56 |
| **LNC15** | 20.88  ±0.61 | 23.50  ±0.62 | 0.33  ±0.01 | 0.33  ±0.02 | -1.17  ±0.07 | -1.2  ±0.39 |
| **LNC17** | 78.89  ±3.33 | 256.5  ±0.19 | 0.25  ±0.03 | 0.60  ±0.11 | -6.09  ±0.28 | -6.1  ±0.36 |
| **LNC20** | 65.53  ±0.58 | 121.5  ±0.15 | 0.28  ±0.001 | 0.52  ±0.05 | -3.89  ±0.12 | -3.34  ±0.38 |
| **LNC23** | 51.22  ±0.72 | 101.6  ±0.91 | 0.45  ±0.01 | 0.55  ±0.001 | -6.84  ±0.05 | -4.79  ±0.4 |
